# Artificial intelligence in neurology, ethics, recent guideline, and law-an Indian perspective

**DOI:** 10.3389/fneur.2025.1515041

**Published:** 2025-04-02

**Authors:** Tithishri Kundu, Mainak Bardhan

**Affiliations:** ^1^Department of Pharmacology, Manipal Tata Medical College, Jamshedpur, Manipal Academy of Higher Education, Manipal, India; ^2^Miami Cancer Institute, Baptist Hospital of Miami, Miami, FL, United States

**Keywords:** artificial intelligence (AI), machine learning, ethics, ICMR guidelines, USA, China, European Union (EU), India

## Introduction

Artificial intelligence (AI), a boon, kept healthcare professionals safe during the COVID-19 era in the form of “Tommy”, the robot nurse in Italy or “Mitra” in India. But continuous involvement of AI in healthcare also brings various challenges, i.e., quality and ownership of the data, belief issues, and ethical challenges. Therefore, in this article, we want to discuss the global policies regarding AI from different countries including India. We also present the summary of the new ethical guideline for AI applications in healthcare by the Indian Council of Medical Research (ICMR) and the Digital Personal Data Protection Act 2023 (DPDP Act), the first law for personal data protection in India, released recently in India.

## Current AI applications in neurology in India

AI can be utilized in different parts of neurology from diagnosis of seizures, developmental anomalies, i.e., Down syndrome, cerebral palsy, etc., and neurodegenerative diseases (e.g., Alzheimer's disease) to the rehabilitation of stroke patients (1). Recently in India, Aster CMI hospital has developed a screening tool to diagnose carpal tunnel syndrome (2). Scientists from Kyoto University, Japan, and IIT Roorkee, India, have created an AI-based model to predict the glioma grading from brain MRI which will help to treat the tumors (3). A USA-based company named Intel and the University of California have partnered with 29 international institutes including Tata Memorial Hospital, Mumbai to create an AI model for early detection of brain tumor (4). Medtronic India has partnered with Qure.ai to develop an AI-based tool for early detection and management of stroke patients (5).

## Ethical laws and guidelines around the world

### USA

Around 120 bills are being contemplated by US Congress regarding AI but significantly less number of bills are related to ethics in healthcare. The Health Insurance Portability and Accountability Act of 1996 (HIPAA) exists in USA for personal data security of the citizens. However, HIPPA cannot shield the citizen from the “Black box” problem of AI. American Medical Association's “Augmented Intelligence in Medicine” (2018) and “Payment and Coverage of AI” (2019), “Blueprint for an AI Bill of Rights” (2022) by the White House, and “Artificial Intelligence Risk Management Framework 1.0 (AI RMF)” (2023) by National Institute of Standards and Technology (NIST), exist regarding AI (6). But these policies cannot protect the citizens completely. Food and Drug Administration (FDA) has several guiding principles regarding AI-enabled medical devices (e.g., DermaSensor and Paige Prostate to detect skin cancer and carcinoma prostate respectively), i.e., “Good Machine Learning Practice for Medical Device Development: Guiding Principles” (2021), “Transparency for Machine Learning-Enabled Medical Devices: Guiding Principles”(2021), “Transparency for Machine Learning-Enabled Medical Devices: Guiding Principles” (2024) (7). But till date no such guideline exists for non-device AI, i.e., AI associated with the clinical decision support system.

### Germany

Similar to HIPPA in the USA, the General Data Protection Regulation (GDPR) is present in the European Union (EU) for the personal data security of citizens. Similarly, as a member of the EU, Bundesdatenschutzgesetz (BDSG) is present in Germany. General Product Safety Regulation (GPSR) 2023/988 involves safety rules for products. The New Product Liability Directive includes compensation for AI software which will be implemented in December 2026. Though the terminology “AI” is included as a reference in the German Works Constitution Act 2021, Germany does not have a separate AI regulation and will probably follow the EU AI Act. Among all the regulations, the EU AI Act (2024) is the most significant legislation as it is the first legal framework for AI. This act divides AI into several risk categories, i.e., unacceptable (e.g., Biometric identification), high (e.g., Facial recognition), limited (e.g., ChatGPT and other chatbots), and low or minimal (e.g., Video games) risk (8). Depending on their risk category, different compliance requirements exist. Whereas, in the unaccepable risk category, the AI will be prohibited from further use, in case of the limited risk category, only a transparency requirement is present. Most of the rules in the EU AI Act will be applicable from August 2026.

### Canada

Canada has several data protection laws. Privacy Act and the Personal Information Protection and Electronic Documents Act (PIPEDA) exist at the federal level and various regulations, i.e., the Personal Information Protection Act, SA 2003 and the Personal Information Protection Act in Alberta and British Columbia, respectively, are present at the provincial level (9). A federal law, Artificial Intelligence and Data Act (AIDA) was initiated in 2022 but hopefully will come into effect soon. AIDA divides the key players into three categories, i.e., developers, deployers, and operators. It is mainly applicable to high-impact AI only and does not have a sector-specific approach. One of the main objectives of AIDA is to promote the safe use of AI. Health Canada, USFDA, and Medicines and Healthcare Products Regulatory Agency (MHRA)-UK, have jointly issued a guiding principle for AI in late 2021 followed by a draft premarket guideline of machine-learning enabled medical devices (MLMD) introduced by Health Canada in 2023.

### China

China has several laws for data protection, i.e., Personal Information Protection Law (PIPL), Data Security Law, etc. There are several policies addressing the development of AI in China, i.e., “Made in China 2025” (2015), Central Committee of the Communist Party of China (CCP)'s 13th five-year plan (2016) etc. (10). Different policies regarding the ethical use of AI are the New Generation Artificial Intelligence Development Plan (AIDP), 2017 regarding ethical aims in AI, the National Governance Committee policy, 2019 regarding the ethical principles of AI, and the Ethics Code in 2021. China's cybersecurity body published “Guidelines for Artificial Intelligence Ethical Security Risk Prevention” in 2021. The First legislative regulation titled “Generative AI Measures” for the development and protection of AI by the Cyberspace Administration of China, Ministry of Industry and Information Technology, Ministry of Science and Technology, and several other ministries is applicable from 2023. China's National Technical Committee published the “AI Safety Governance Framework” in September 2024 which categorizes the types of risk by AI into two types and six subtypes including ethical risk. But this framework only describes the types of risk whereas the European Union's risk categorization divides the risk and the associated legislations related to AI into four categories. Overall, the AI policies in China are development-centric compared to the European Union's policy to shield basic human rights.

## Indian laws and policies

**Figure 1:**
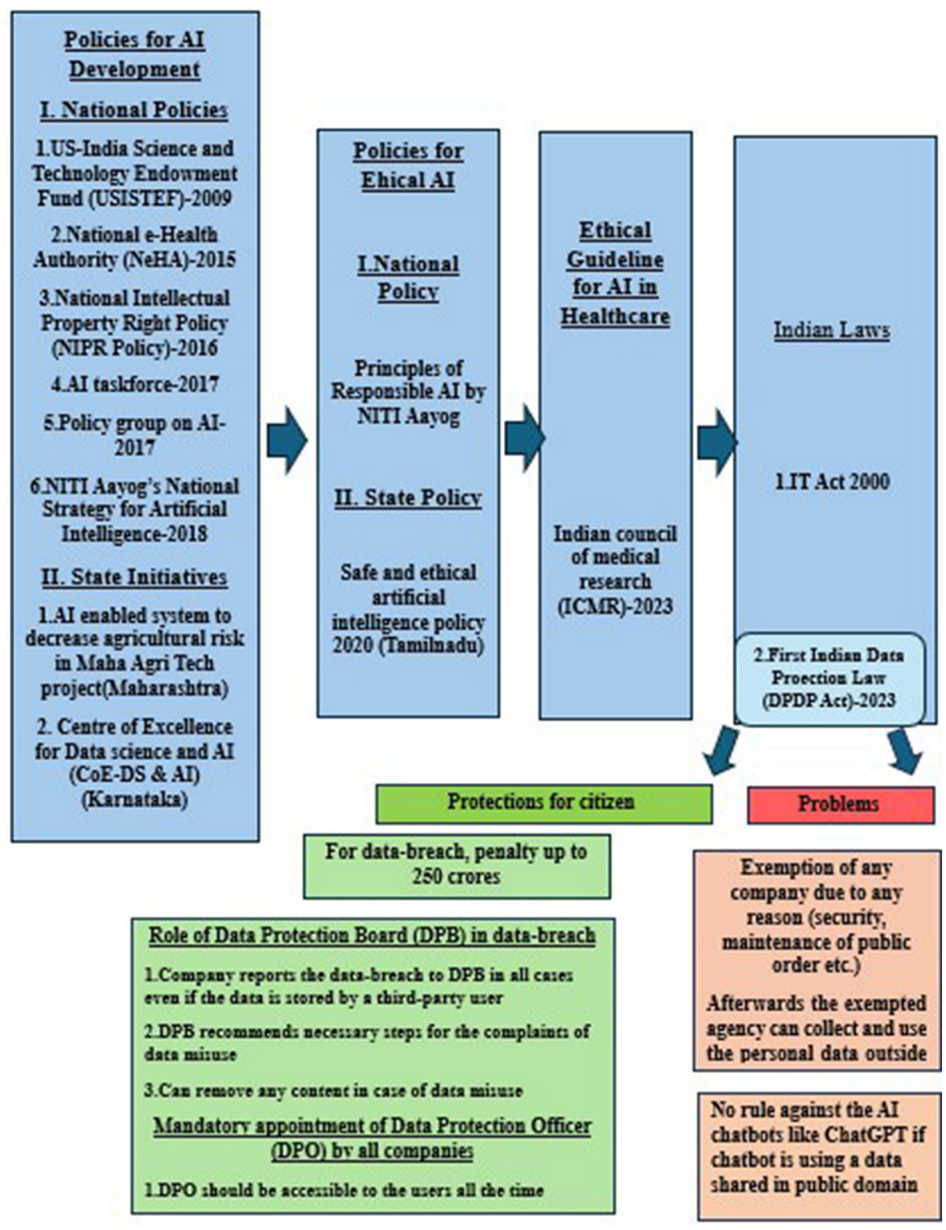
Continuous progression of Indian policies and guidelines regarding AI.

As AI applications in healthcare evolve, newer policies and guidelines are needed. Several policies and guidelines exist for the development and ethical use of AI in India (11, 12). However, none of these policies adheres explicitly to the ethical issues of AI in biomedical research. ICMR is a national organization that provides ethical guidelines for biomedical research on human participants in India. This recent guideline by ICMR is unique as it describes the ethical guideline of the application of AI in healthcare. Till 2023, only the IT Act 2000 existed in India for data protection, which states that the corporate organization dealing with the data is liable for compensation. In August 2023, the Indian government announced the DPDP Act (13), the first law for personal data protection in India. Details of the continuous progression of Indian policies and guidelines are depicted in Figure 1.

## Ethical issues addressed by new Indian ICMR guideline-“Human in the Loop” (HITL) model, accountability, pre-deployment testing, use of native language, informed consent-pre-consent-reconsent, and “Right to be Forgotten”

One valid concern regarding the application of AI in healthcare is that it might threaten human autonomy as the decision-making is the responsibility of the machines. ICMR guideline specifies the inclusion of the “Human in the Loop” (HITL) model in AI, i.e., if necessity arises, humans can override the device and should be capable of decision-making (14). For example, AI can diagnose potential malignancy in PET scans. However, clinicians should not accept AI's decision blindly. They should know how well AI performs in detecting this specific type of malignancy in a patient of the same gender, race, etc., and reach a diagnosis. Another issue that the guideline addressed is regarding accountability and liability. The guideline says that the responsibility for glitches in AI rests on the nature of the flaws. If the malfunction is due to development issues, the responsibility lies with the developer. On the other hand, if the fault is related to execution, the end-user is accountable for it. For instance, if AI calculates the wrong dose of insulin and the patient has hypoglycemia, the responsibility lies with the manufacturer. But if the clinician gives wrong input, leading to the miscalculation of insulin dose, the clinician is accountable.

One of the valid concerns regarding AI is the quality and clinical validation of the data. AI applications in healthcare are based on data. AI is fed with a dataset as input and provides us with decisions or output. But the argument is that the verdict delivered by AI is based on the “Training Data”, i.e., the data provided to AI during the development phase. So, is the decision delivered by AI valid? As a solution to this problem, the ICMR guideline mentions that first, the “Training Data” of AI should have an adequate sample size and represent the population, including ethnic minorities. Then, the data should go through clinical validation, i.e., the application of AI in real-world clinical scenarios. “Pre-deployment Testing” should also be carried out in every new site where AI will be applied. Another problem with AI is that in Lower- and Middle-Income countries (LMIC) like India, technology is not available to everybody, and thus exists a “Digital Divide”. To resolve the issue, the ICMR guideline states that AI technology should be accessible and equally distributed to all. Also, the user interface of AI should use native languages to conquer the language barrier. ICMR guideline stresses that during the AI development phase, informed consent, pre-consent, and re-consent should be taken from the participants. Patients should have the “Right to be Forgotten”, i.e., patients' data should be excluded or modified in case they opt out later.

## Ethical review process of AI protocol in Institutional Ethics Committee (IEC) by new Indian ICMR guideline

Similar to any other country, in India, before undergoing any research, the researchers need IEC permission. The new guideline states that the IEC, constantly involved in AI technology, should have specific subject experts, i.e., experts in AI technology, legal experts, etc., in the committee. Also, as an additional measure, ethics committee members should undergo constant training related to data analytics, machine learning, etc.

## Conclusion

The benefit or harm due to AI rests in our hands. With the continuous expansion of AI, policies, and guidelines should also be evolved. This article summarizes the policies regarding AI in several countries, i.e., USA, Canada, Germany and China. This article also describes the Indian laws and policies regarding the development and ethical use of AI. Though there are several policies in India regarding AI, none adheres explicitly to the ethical issues of AI in biomedical research. This recent guideline described by the authors is unique as it represents the ethical guideline of the application of AI in healthcare and the first law for personal data protection in India (DPDP Act 2023).

## References

[1] Surianarayanan C Lawrence JJ Chelliah PR Prakash E Hewage C. Convergence of artificial intelligence and neuroscience towards the diagnosis of neurological disorders—a scoping review. Sensors. (2023) 23:3062. 10.3390/s23063062 36991773 PMC10053494

[2] Ang A. AIIMS Delhi Unveils New Oncology AI for Early Cancer Detection and More AI Briefs From India. Healthcare IT News. Asia: Artificial intelligence (2024). Available online at: https://www.healthcareitnews.com/news/asia/aiims-delhi-unveils-new-oncology-ai-early-cancer-detection-and-more-ai-briefs-india#:~:text=Artificial%20Intelligence-,AIIMS%20Delhi%20unveils%20new%20oncology%20AI%20for%20early%20cancer%20detection,for%20detecting%20Carpal%20Tunnel%20Syndrome [accessed January 28, 2025).

[3] Indian Scientists Develop AI to Enhance Brain Tumour Diagnosis. Business Insider India. Home: Science (2020). Available online at: https://www.businessinsider.in/science/news/indian-scientists-develop-ai-to-enhance-brain-tumour-diagnosis/articleshow/76283617.cms (accessed January 28, 2025).

[4] Intel to Develop Technology to Train AI Help Identify Brain. Times of India. News: Gadgets News (2020). Available online at: https://timesofindia.indiatimes.com/ gadgets-news/intel-to-develop-technology-to-train-ai-help-identify-brain-tumors/articleshow/75695636.cms (accessed January 28, 2025).

[5] Medtronic Partners With Qure.ai to Advance Stroke Management Using Artificial Intelligence in India. The Financial Express. Business News: Healthcare: Healthtech (2023). Available online at: https://www.financialexpress.com/healthcare/healthtech/medtronic-partners-with-qure-ai-to-advance-stroke-management-using-artificial-intelligence-in-india/3033613/(accessed January 28, 2025).

[6] American Medical Association. Principles for Augmented Intelligence Development, Deployment, and Use. (2023). Availabler online at: http://www.~ama-assn.org/system/files/ama-ai-principles (accessed January 28, 2025).

[7] U.S Food and Drug Administration. Artificial Intelligence and Machine Learning in Software as a Medical Device. Available online at: https://www.fda.gov/medical-devices/software-medical-device-samd/artificial-intelligence-and-machine-learning-software-medical-device (accessed January 28, 2025).

[8] Busch F Kather JN Johner C Moser M Truhn D Adams LC. Navigating the European union artificial intelligence act for healthcare. NPJ Digital Med. (2024) 7:210. 10.1038/s41746-024-01213-6 39134637 PMC11319791

[9] White & Case LLP.AI Watch: Global regulatory tracker – Canada. USA: White & Case LLP (2024). Available online at: https://www.whitecase.com/insight-our-thinking/ai-watch-global-regulatory-tracker-canada (accessed January 28, 2025).

[10] Roberts H Cowls J Hine E Morley J Wang V Taddeo M. Governing artificial intelligence in China and the European Union: Comparing aims and promoting ethical outcomes. Inform Soc. (2023) 39:79–97. 10.1080/01972243.2022.2124565

[11] Chatterjee S Dohan MS. Artificial intelligence for healthcare in India: policy initiatives, challenges, and recommendations. Int J Healthcare Inform Syst Inform. (2021) 16:1–1. 10.4018/IJHISI.20211001.oa1740124859

[12] NITI Aayog. Responsible AI. Approach Document for India Part 1 – Principles for Responsible AI. Available online at: https://www.niti.gov.in/sites/default/files/2021-02/Responsible-AI-22022021.pdf (accessed January 28, 2025).

[13] Ministry of Electronics and Information Technology. Digital and Personal Data Protection Act 2023. Available online at: https://www.meity.gov.in/writereaddata/files/Digital%20Personal%20Data%20Protection%20Act%202023.pdf (accessed January 28, 2025).

[14] Indian Council of Medical Research (ICMR). Ethical Guidelines for Application in Artificial Intelligence in Biomedical Research and Healthcare. (2023). Available online at: https://main.icmr.nic.in/content/ethical-guidelines-application-artificial-intelligence-biomedical-research-and-healthcare (accessed January 28, 2025).

